# Reduced All-Cause Mortality in the ETHOS Trial of Budesonide/Glycopyrrolate/Formoterol for Chronic Obstructive Pulmonary Disease. A Randomized, Double-Blind, Multicenter, Parallel-Group Study

**DOI:** 10.1164/rccm.202006-2618OC

**Published:** 2021-03-01

**Authors:** Fernando J. Martinez, Klaus F. Rabe, Gary T. Ferguson, Jadwiga A. Wedzicha, Dave Singh, Chen Wang, Kimberly Rossman, Earl St. Rose, Roopa Trivedi, Shaila Ballal, Patrick Darken, Magnus Aurivillius, Colin Reisner

**Affiliations:** ^1^ Joan and Sanford I. Weill Department of Medicine, Weill Cornell Medicine, New York, New York; ^2^ LungenClinic Grosshansdorf and Christian-Albrechts University Kiel, Airway Research Center North, Member of the German Center for Lung Research (DZL), Grosshansdorf, Germany; ^3^ Pulmonary Research Institute of Southeast Michigan, Farmington Hills, Michigan; ^4^ National Heart and Lung Institute, London, United Kingdom; ^5^ Medicines Evaluation Unit, University of Manchester, Manchester University NHS Foundation Hospitals Trust, Manchester, United Kingdom; ^6^ National Clinical Research Centre for Respiratory Diseases, China-Japan Friendship Hospital, Beijing, China; ^7^ AstraZeneca, Morristown, New Jersey; ^8^ AstraZeneca, Durham, North Carolina; ^9^ AstraZeneca, Wilmington, Delaware; and; ^10^ AstraZeneca, Gothenburg, Sweden

**Keywords:** BGF metered-dose inhaler, chronic obstructive pulmonary disease, inhaled corticosteroids/long-acting muscarinic antagonist/long-acting β_2_-agonist, mortality, triple therapy

## Abstract

**Rationale:** In the phase III, 52-week ETHOS (Efficacy and Safety of Triple Therapy in Obstructive Lung Disease) trial in chronic obstructive pulmonary disease (COPD) (NCT02465567), triple therapy with budesonide/glycopyrrolate/formoterol fumarate (BGF) significantly reduced all-cause mortality compared with glycopyrrolate/formoterol fumarate (GFF). However, 384 of 8,509 patients were missing vital status at Week 52 in the original analyses.

**Objectives:** To assess the robustness of the ETHOS mortality findings after additional data retrieval for patients missing Week 52 vital status in the original analyses.

**Methods:** Patients with moderate to very severe COPD and prior history of exacerbation received twice-daily dosing with 320/18/9.6 μg of BGF (BGF 320), 160/18/9.6 μg of BGF (BGF 160), 18/9.6 μg of GFF, or 320/9.6 μg of budesonide/formoterol fumarate (BFF) (all delivered via a single metered-dose Aerosphere inhaler). Time to death (all-cause) was a prespecified secondary endpoint.

**Measurements and Main Results:** In the final retrieved dataset, which included Week 52 vital status for 99.6% of the intent-to-treat population, risk of death with BGF 320 was significantly lower than GFF (hazard ratio, 0.51; 95% confidence interval, 0.33–0.80; unadjusted *P* = 0.0035). There were no significant differences in mortality when comparing BGF 320 with BFF (hazard ratio, 0.72; 95% confidence interval, 0.44–1.16; *P* = 0.1721), nor were significant differences observed when comparing BGF 160 against either dual comparator. Results were similar when the first 30, 60, or 90 days of treatment were excluded from the analysis. Deaths from cardiovascular causes occurred in 0.5%, 0.8%, 1.4%, and 0.5% of patients in the BGF 320, BGF 160, GFF, and BFF groups, respectively.

**Conclusions:** Using final retrieved vital status data, triple therapy with BGF 320 reduced the risk of death compared with GFF, but was not shown to significantly reduce the risk of death compared with BFF, in patients with COPD. Triple therapy containing a lower dose of inhaled corticosteroid (BGF 160) was not shown to significantly reduce the risk of death compared with the dual therapy comparators.

**At a Glance Commentary**

**Scientific Knowledge on the Subject**

The ETHOS trial in patients with chronic obstructive pulmonary disease (COPD) found that triple therapy with 320/18/9.6 μg budesonide/glycopyrrolate/formoterol metered-dose inhaler, an inhaled corticosteroid (ICS)/long-acting muscarinic antagonist (LAMA)/long-acting β_2_-agonist (LABA) combination, reduced the risk of all-cause mortality compared with LAMA/LABA therapy.

**What This Study Adds to the Field**

Additional analyses of mortality, including final retrieved vital status data, demonstrated that these findings were robust and were not due solely, or even primarily, to an acute ICS withdrawal effect. Furthermore, adjudicated causes of death and results for the time from exacerbation to death suggest a potential role for ICS in mortality that may not be directly related to effects on COPD exacerbations. Our findings underscore the need to target mortality reduction as an achievable goal in the treatment of COPD.

Chronic obstructive pulmonary disease (COPD) is the third leading cause of death globally (1). Pharmacological treatments for COPD include bronchodilators (long-acting muscarinic antagonist [LAMA] and/or long-acting β_2_-agonist [LABA]), which may be combined with an inhaled corticosteroid (ICS) (2). These medications improve lung function and symptoms and reduce the frequency of COPD exacerbations; however, to date, clinical trial data have provided inconsistent evidence of benefits on mortality (2). The LAMA tiotropium decreased mortality versus placebo during 4 years of treatment in patients with moderate to very severe COPD (3). In addition, two long-term trials that assessed ICS/LABA therapy in moderate or moderate to very severe COPD failed to demonstrate a significant difference in all-cause mortality versus placebo despite trends in favor of the active treatments (4, 5).

Although assessing mortality was not the primary objective, two recent phase III trials of inhaled triple combination therapy (ICS/LAMA/LABA) in COPD, ETHOS (Efficacy and Safety of Triple Therapy in Obstructive Lung Disease) and IMPACT (Informing the Pathway of COPD Treatment), have suggested a beneficial effect of triple therapy on all-cause mortality compared with LAMA/LABAs (6, 7). All-cause mortality was a secondary endpoint in ETHOS, which evaluated triple therapy at two different ICS doses (320/18/9.6 μg and 160/18/9.6 μg of budesonide/glycopyrrolate/formoterol fumarate [BGF] metered-dose inhaler; hereafter referred to as BGF 320 and BGF 160, respectively) versus dual therapy with glycopyrrolate/formoterol fumarate (GFF) metered-dose inhaler (LAMA/LABA) or budesonide/formoterol fumarate (BFF) metered-dose inhaler (ICS/LABA) (7). For the all-cause mortality endpoint in ETHOS, which included deaths that occurred on and off treatment, there was a 46% risk reduction with BGF 320 (and a nonsignificant 21% reduction with BGF 160) versus GFF, suggesting a possible dose–response effect of ICS therapy on mortality. Similarly, in the IMPACT trial, which assessed all-cause mortality as a prespecified other endpoint, there was a 29% risk reduction with 100/62.5/25 μg of fluticasone furoate/umeclidinium/vilanterol (ICS/LAMA/LABA) versus 62.5/25 μg of umeclidinium/vilanterol (LAMA/LABA), including on- and off-treatment deaths (6).

Of the 8,509 patients in the ETHOS intent-to-treat (ITT) population, 384 patients were missing vital status data at Week 52. Given the clinical importance of this endpoint, it was necessary to assess the mortality findings from ETHOS thoroughly. Here, we present additional analyses evaluating the reduction in all-cause mortality with BGF relative to GFF in ETHOS to assess the robustness of the effect, the possible impact of ICS withdrawal, and the relationship between COPD exacerbations and mortality. These analyses were performed after the collection of additional vital status information after trial completion for patients with incomplete vital status at the end of the study. Some of these data have been previously presented in the form of an abstract (8).

## Methods

### Study Design

Details of the study design have been published (7, 9). Briefly, ETHOS (NCT02465567) was a 52-week, randomized, double-blind, parallel-group trial conducted across 26 countries. Patients received twice-daily dosing with BGF 320, BGF 160, GFF 18/9.6 μg, or BFF 320/9.6 μg. All treatments were delivered orally from a single metered-dose Aerosphere inhaler (AstraZeneca); doses represent the sum of two actuations.

Eligible patients were 40–80 years of age with symptomatic COPD (COPD Assessment Test score ≥10 at screening despite receiving two or more inhaled maintenance therapies), a post-bronchodilator FEV_1_ 25–65% of predicted normal, a smoking history ≥10 pack-years, and a documented history of ≥1 moderate or severe COPD exacerbations in the previous year (if their FEV_1_ was <50% of predicted) or ≥2 moderate or ≥1 severe COPD exacerbations (if their FEV_1_ was ≥50% of predicted). Patients with a current diagnosis of asthma or significant diseases other than COPD (including other respiratory conditions, cardiac disease, and cancer) were excluded (7, 9).

### Endpoints and Assessments

The primary endpoint of the trial (not reported in this manuscript) was the rate of moderate or severe COPD exacerbations (7). Time to death (all-cause) was a prespecified secondary endpoint that was assessed in the ITT population using the treatment policy estimand, which included all randomized patients who received any amount of study drug and all observed data regardless of whether patients remained on randomized treatment.

In the original dataset, 384 patients had incomplete vital status at database lock. Vital status was recorded for the majority of these patients; however, the collection of this data occurred before Week 52. Subsequently, clinical sites were asked to contact patients or next of kin, search public records (e.g., obituaries, death registries, and voter registration), determine whether patients had visited primary care provider offices or local hospitals, and conduct searches on social media (where permitted by local privacy regulations). A vendor who specializes in obtaining missing vital status information (OmniTrace Corporation) was also engaged to assist in this process. A final retrieved dataset was created including this additional Week 52 vital status information; the original database that was established at database lock was left unchanged.

Using the final retrieved dataset, the following analyses of the time to death (all-cause) were performed: on- and off-treatment deaths, on-treatment deaths only, and on- and off-treatment deaths by subgroups according to prior exacerbation history, FEV_1_% predicted, and prior medications. The relationship between COPD exacerbations and mortality was assessed by analyzing moderate or severe exacerbation rates and severe exacerbation rates according to vital status, and the median time from exacerbation to death. The robustness of the results was explored using tipping-point analyses, in which missing vital status data for patients in the BGF 320 and GFF groups were imputed according to various scenarios. Two tipping-point analyses imputed the hazard rates for BGF 320 and GFF incrementally until the tipping point was reached (i.e., when the *P* value of the comparison of BGF 320 versus GFF was >0.046 [the critical level of significance in ETHOS, after adjustment due to the interim analysis]) (7). A third tipping-point analysis imputed the vital status of the patients with missing data; those in the GFF group were imputed as alive, and the number of deaths in the BGF 320 group was increased incrementally to determine whether the critical level of significance would still be reached. Landmark analyses of time to death were conducted to assess the possible impact of ICS withdrawal on the findings by excluding the first 30, 60, or 90 days of treatment and by assessing the hazard ratio (HR) throughout the study, excluding all previous data. All time-to-death analyses used a Cox regression model adjusting for baseline post-bronchodilator FEV_1_% predicted and age. *P* values for all analyses were not adjusted for multiplicity. Causes of death were adjudicated, when available, by an independent clinical endpoint committee.

## Results

### Study Population

The ITT population included 8,509 patients (mean age, 64.7 yr; 59.7% male; mean post-bronchodilator FEV_1_, 43.4% of predicted normal value). Full baseline demographics have been previously published (7). Overall, 56.5% had experienced two or more moderate or severe exacerbations in the previous year, 59.9% had a blood eosinophil count ≥150 cells/mm^3^, and 80.5% were using ICS at screening. The distribution of cardiovascular risk factors was similar across treatment groups (Table E1 in the online supplement).

Supplemental data collection confirmed vital status for 354/384 patients who had unknown vital status at Week 52 in the original dataset, leaving only 30 patients with unknown Week 52 vital status in the final retrieved dataset (*n* = 5–10 across groups), representing 0.4% of the ITT population.

### Time to Death (All-Cause)

**Figure 1.**
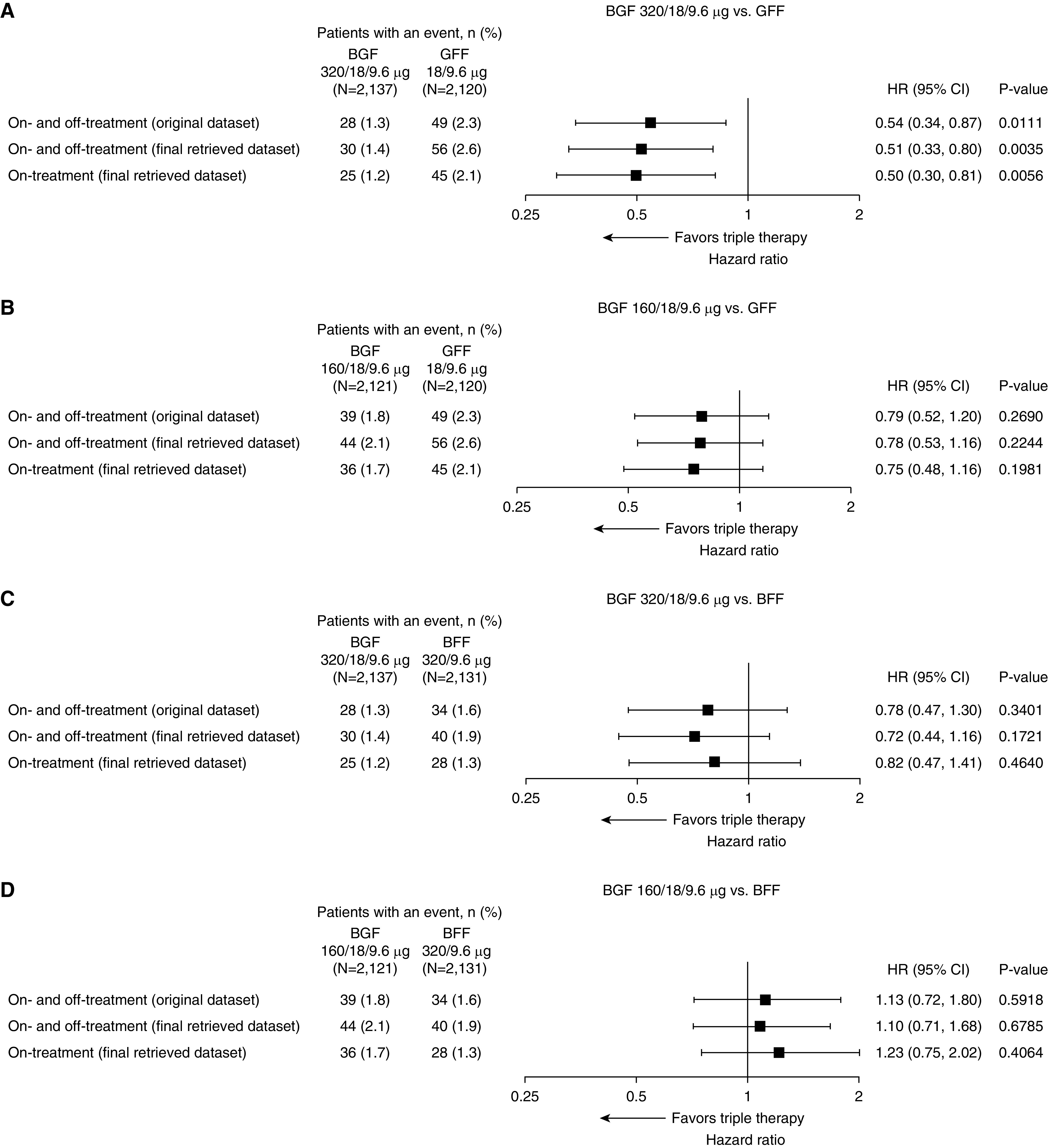
Forest plots for time to death (all-cause) for (*A*) 320/18/9.6 μg budesonide/glycopyrrolate/formoterol fumarate (BGF) versus glycopyrrolate/formoterol fumarate (GFF), (*B*) 160/18/9.6 μg BGF versus GFF, (*C*) 320/18/9.6 μg BGF versus budesonide/formoterol fumarate (BFF), and (*D*) 160/18/9.6 μg BGF versus BFF (intent-to-treat population). Results for the original dataset are from Reference 7. Significant *P* values (i.e., <0.046) in the original dataset are unadjusted because of an endpoint in the type I error control testing hierarchy not reaching significance. The on-treatment analysis includes deaths that occurred within 30 days of the last day of treatment. The intent-to-treat population included all patients who were randomized to treatment and received any amount of study drug. CI = confidence interval; HR = hazard ratio.

An overview of the results from the original dataset (on- and off-treatment deaths), the final retrieved dataset (on- and off-treatment deaths), and the final retrieved dataset (on-treatment deaths only) is provided in Figure 1.

**Figure 2.**
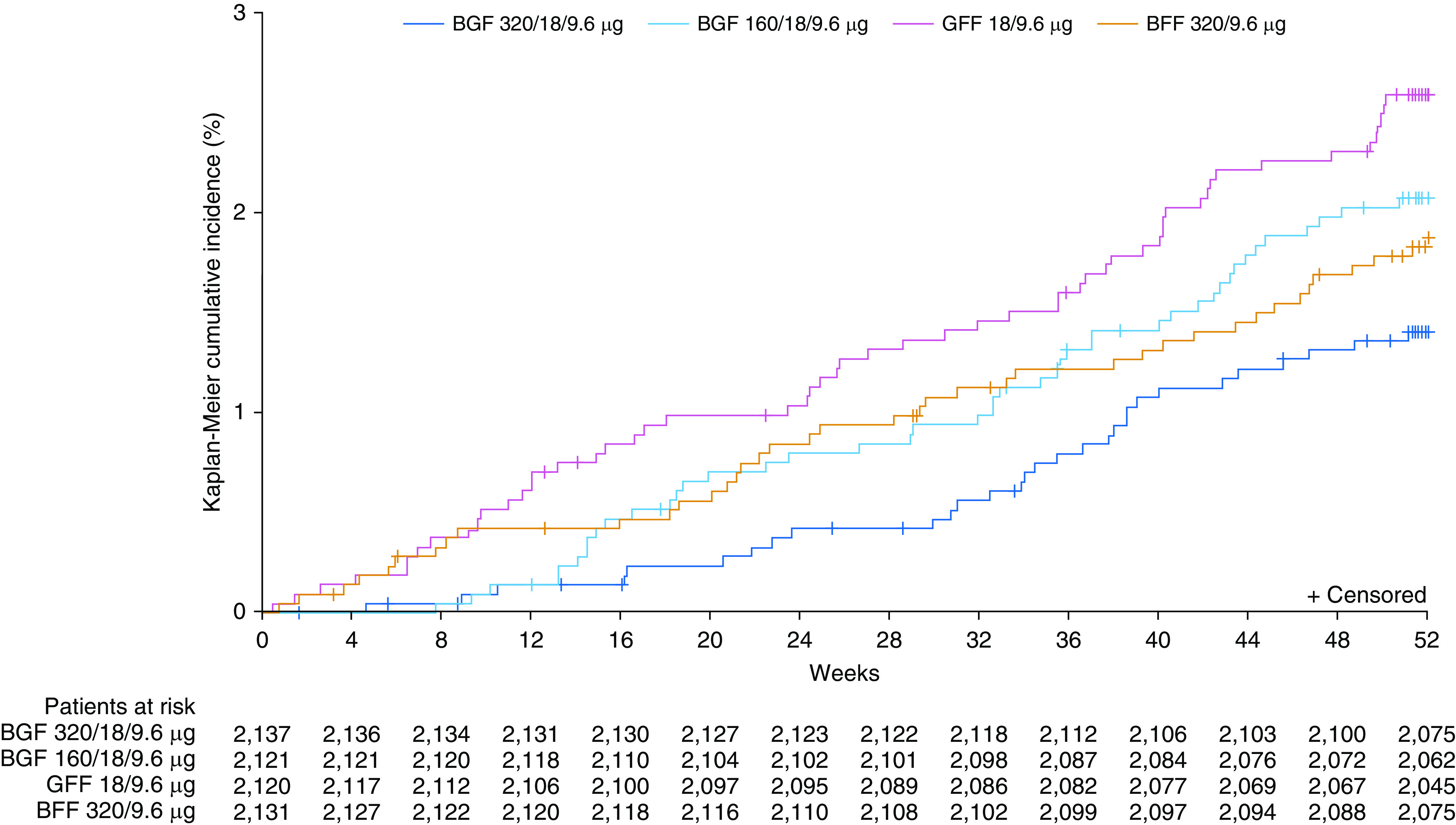
Kaplan-Meier plot for time to all-cause death (final retrieved dataset; intent-to-treat population). BFF = budesonide/formoterol fumarate; BGF = budesonide/glycopyrrolate/formoterol fumarate; GFF = glycopyrrolate/formoterol fumarate.

As in the original dataset (7), the risk of death on and off treatment (all-cause) in the final retrieved dataset was significantly lower with BGF 320 relative to GFF (HR, 0.51; 95% confidence interval [CI], 0.33–0.80; 49% reduction; unadjusted *P* = 0.0035), equivalent to a number needed to treat of 80 (95% CI, 58–198). BGF 320 did not significantly lower the risk of death relative to BFF, although there was a trend in favor of BGF 320 (HR, 0.72; 95% CI, 0.44–1.16; 28% reduction; *P* = 0.1721); there was also a trend for BGF 320 versus BGF 160 (HR, 0.66; 95% CI, 0.41–1.05; 34% reduction; *P* = 0.0766) (Figures 1 and 2). The risk of death was also lower (though not significantly) with BGF 160 relative to GFF (HR, 0.78; 95% CI, 0.53–1.16; 22% reduction; *P* = 0.2244) and was similar for BGF 160 relative to BFF (Figures 1 and 2). Two analyses were performed including only on-treatment data, defining on-treatment deaths either as those that occurred within *1*) 30 days or *2*) 7 days from the last day of treatment. Findings from both analyses were similar to the on- and off-treatment results (Figure 1 and Table E2).

### Tipping-Point Analyses of Time to Death (All-Cause)

To assess the possible impact of the data from the 30 patients with missing Week 52 vital status in the final retrieved dataset, three tipping-point analyses were performed for the comparison of BGF 320 with GFF. Two tipping-point analyses using imputation of hazard rates showed that the postwithdrawal hazards for BGF 320 would have to be more than 230 and 227 times higher than prewithdrawal hazards, respectively, for the comparison with GFF to lose significance in the final retrieved dataset (Figures E1 and E2), a highly unlikely scenario. In contrast, for the equivalent tipping-point analyses in the original dataset, postwithdrawal hazards for BGF 320 would have to be 6–7 times higher than prewithdrawal hazards for the comparison with GFF to lose significance.

In a third tipping-point analysis, various survival assumptions were made for the 30 patients who were missing Week 52 vital status in the final retrieved dataset, of whom 10 received BGF 320 and 5 received GFF. If all 5 patients who received GFF were alive at Week 52, and 8/10 patients in the BGF 320 group died the day after censoring, the comparison between BGF 320 and GFF would remain significant (HR, 0.65; 95% CI, 0.43–0.99; unadjusted *P* = 0.0454).

### Landmark and Subgroup Analyses of Time to Death (All-Cause)

**Figure 3.**
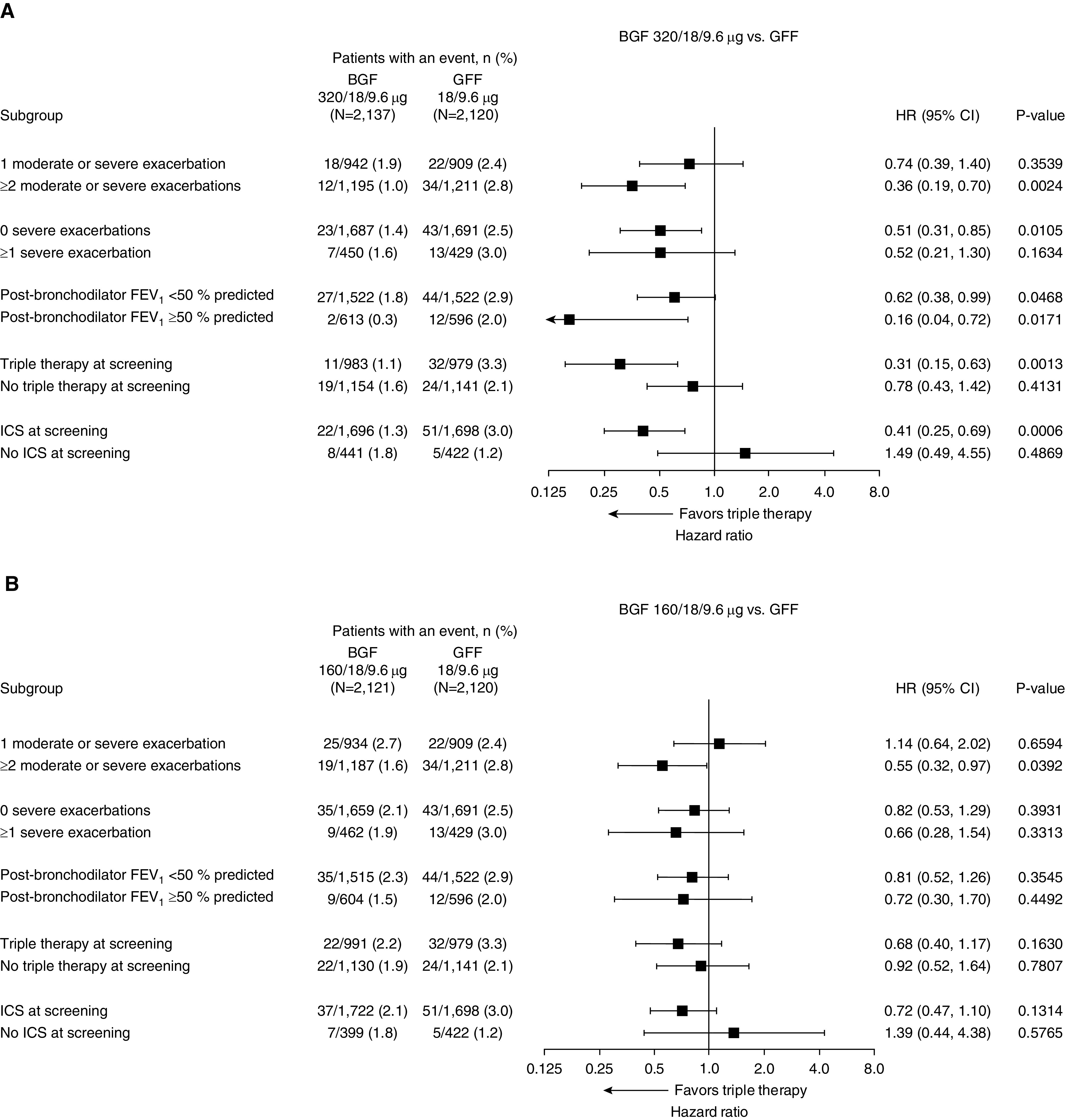
Forest plots for subgroup analyses of time to death (all-cause) for (*A*) 320/18/9.6 μg budesonide/glycopyrrolate/formoterol fumarate (BGF) versus glycopyrrolate/formoterol fumarate (GFF) and (*B*) 160/18/9.6 μg BGF versus GFF (final retrieved dataset; intent-to-treat population). CI = confidence interval; HR = hazard ratio; ICS = inhaled corticosteroid.

**Figure 4.**
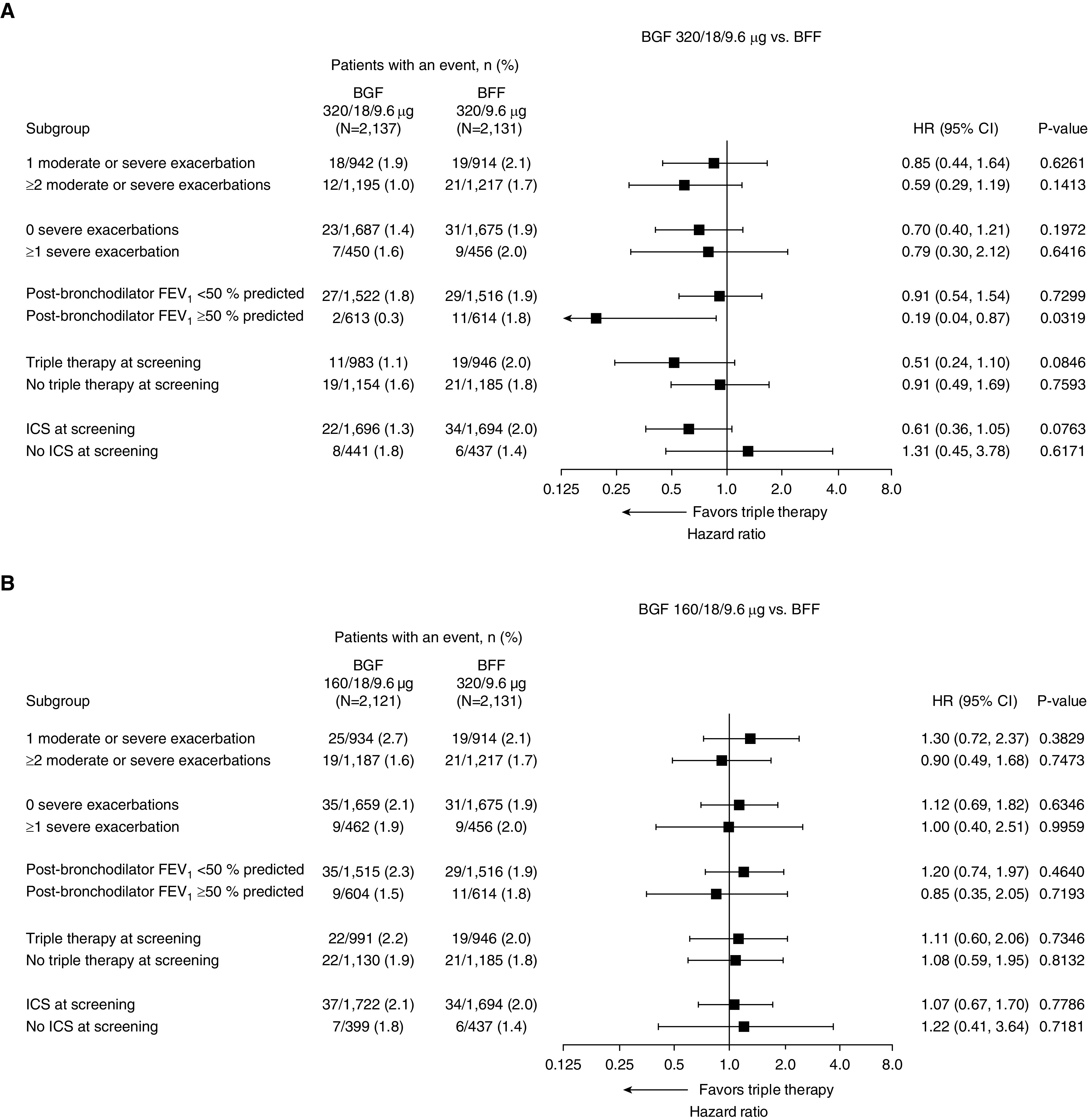
Forest plots for subgroup analyses of time to death (all-cause) for (*A*) 320/18/9.6 μg budesonide/glycopyrrolate/formoterol fumarate (BGF) versus budesonide/formoterol fumarate (BFF) and (*B*) 160/18/9.6 μg BGF versus BFF (final retrieved dataset; intent-to-treat population). CI = confidence interval; HR = hazard ratio; ICS = inhaled corticosteroid.

Subgroup analyses were performed using the final retrieved dataset to assess the benefit of triple therapy according to various patient characteristics. Numerical benefits for the time to death favoring BGF 320 versus dual therapies were shown across all subgroups, with the exception of the subgroup of patients not receiving ICS at screening (Figures 3 and 4). However, several of these subgroups had a small number of events, including the group with no prior ICS use (*n* = 5–8 deaths across groups), and therefore these results should be interpreted with caution. The numerical benefits of BGF 320 versus dual therapies were largest in patients with two or more exacerbations in the previous year or post-bronchodilator FEV_1_ ≥50% predicted and in those with prior triple therapy or ICS use.

**Figure 5.**
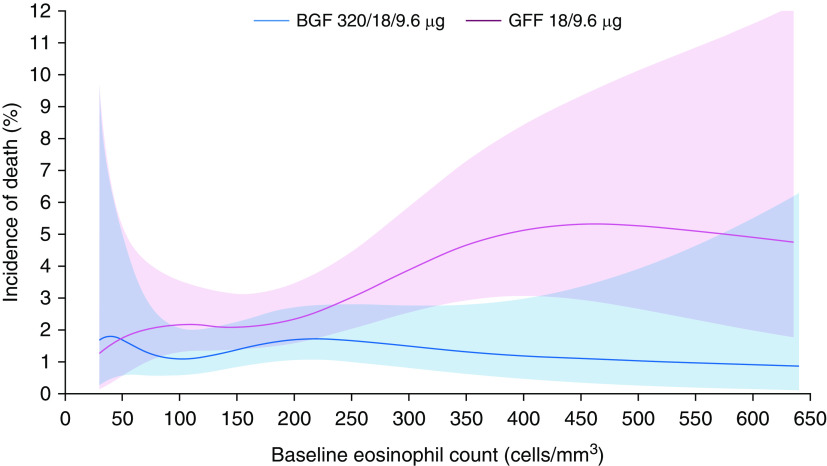
Incidence of death by baseline blood eosinophil count for 320/18/9.6 μg BGF versus GFF (final retrieved dataset; intent-to-treat population). Data are from a generalized additive model. Banded areas indicate 95% CIs that reflect the skewed distribution of eosinophil counts, (i.e., 17.3% of patients had counts <100 cells/mm^3^, 67.9% had 100–300 cells/mm^3^, and 14.7% had >300 cells/mm^3^). BGF = budesonide/glycopyrrolate/formoterol fumarate; CI = confidence interval; GFF = glycopyrrolate/formoterol fumarate.

The incidence of death was also analyzed according to blood eosinophil count as a continuous variable. The benefit of BGF 320 versus GFF in reducing mortality generally increased with eosinophil count (Figures 5 and E3).

**Figure 6.**
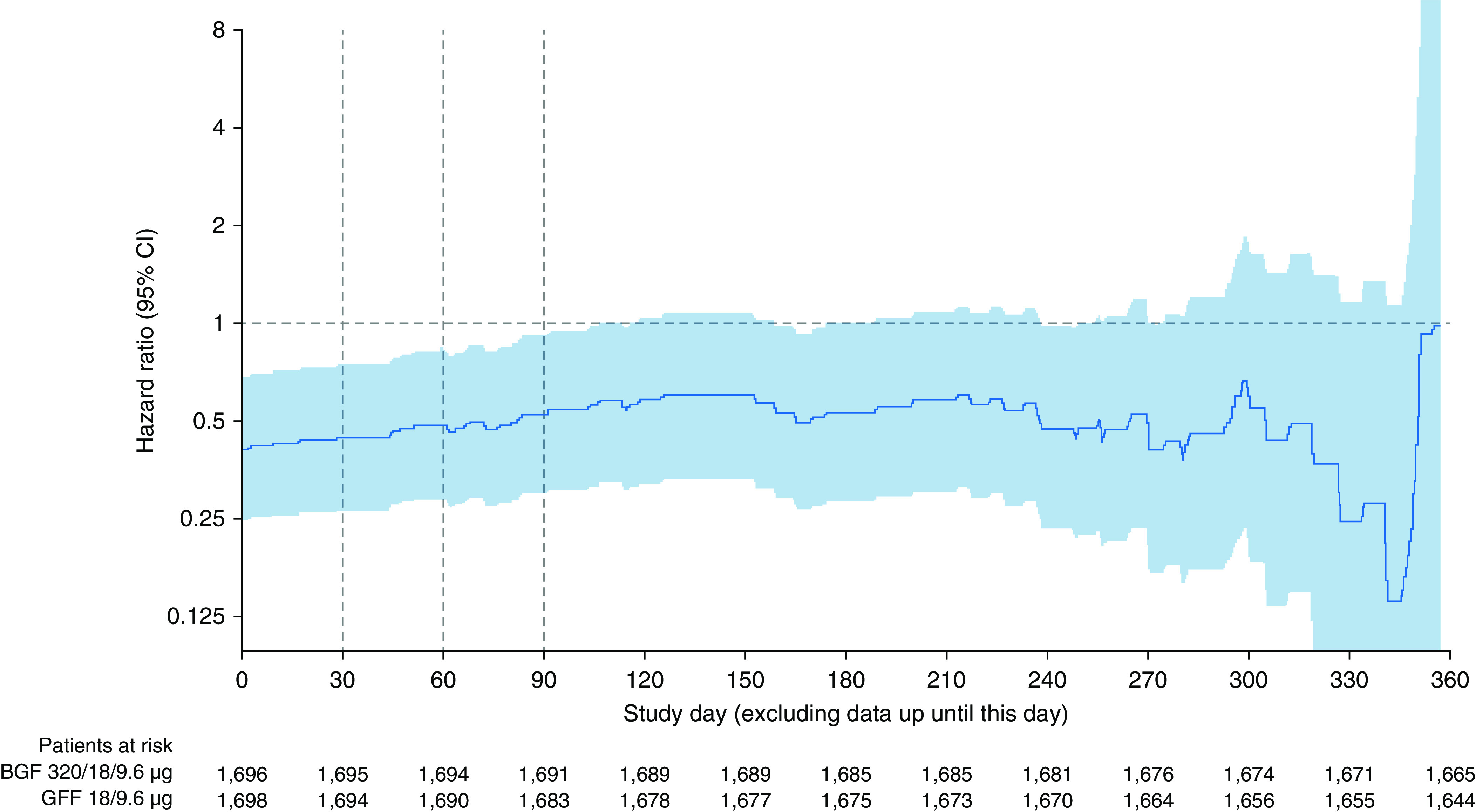
Hazard ratio for time to all-cause death for 320/18/9.6 μg BGF versus GFF over the study period, excluding all previous data, in patients using inhaled corticosteroids at study entry (final retrieved dataset; intent-to-treat population). BGF = budesonide/glycopyrrolate/formoterol fumarate; CI = confidence interval; GFF = glycopyrrolate/formoterol fumarate.

Landmark analyses of time to death were conducted (excluding deaths that occurred in the first 30, 60, or 90 d of the study) to assess the possible impact of acute treatment withdrawal on the findings. Results were similar to those of the full analysis, with reductions in mortality for BGF 320 versus GFF observed in all three analyses (HR ≤ 0.63 for all; Table 1). As in the full analysis, there were reductions in the risk of death for BGF 320 versus BFF and BGF 160, although the differences were not significant. These landmark analyses were also repeated in the subgroup of patients who were using ICS at study entry. Reductions in the risk of mortality for BGF 320 versus GFF were also observed in this subgroup when the first 30, 60, or 90 days of treatment were excluded from the analyses (HR ≤ 0.53 for all; Table E3 and Figure E4). In addition to the landmark analyses at 30, 60, and 90 days, analyses were conducted to evaluate the HR for BGF 320 relative to GFF throughout the study period (excluding all previous events) in the subgroup of patients who were using ICS at study entry (Figure 6) and the full ITT population (Figure E5). The HR for BGF 320 versus GFF remained consistently <1, although the 95% CIs became wider as less data and fewer events remained. There was a risk reduction of at least 34% (prior ICS subgroup) or 27% (overall population) seen throughout the study up to Day 350 (Figures 6 and E5), indicating a robust risk reduction that was not driven by an early period of acute withdrawal effects.

**Table 1.** Time to Death (All-Cause) Occurring after the First 30, 60, or 90 Days of Treatment (Final Retrieved Dataset; ITT Population)

	BGF 320/18/9.6 μg (*N = 2,137*)	BGF 160/18/9.6 μg (*N = 2,121*)	GFF 18/9.6 μg (*N = 2,120*)	BFF 320/9.6 μg (*N = 2,131*)
After 30 d				
*N*	2,136	2,121	2,116	2,126
Patient deaths, *n* (%)	30 (1.4)	44 (2.1)	52 (2.5)	36 (1.7)
BGF 320/18/9.6 μg vs. comparators				
Hazard ratio (95% CI)	—	0.65 (0.41–1.05)	0.55 (0.35–0.87)	0.80 (0.49–1.30)
*P* value		0.0755	0.0103	0.3573
BGF 160/18/9.6 μg vs. comparators				
Hazard ratio (95% CI)	—	—	0.84 (0.57–1.26)	1.22 (0.78–1.89)
*P* value			0.4072	0.3845
After 60 d				
*N*	2,134	2,120	2,112	2,121
Patient deaths, *n* (%)	29 (1.4)	43 (2.0)	48 (2.3)	32 (1.5)
BGF 320/18/9.6 μg vs. comparators				
Hazard ratio (95% CI)	—	0.65 (0.40–1.04)	0.58 (0.36–0.92)	0.86 (0.52–1.43)
*P* value		0.0720	0.0206	0.5710
BGF 160/18/9.6 μg vs. comparators				
Hazard ratio (95% CI)	—	—	0.89 (0.59–1.35)	1.34 (0.85–2.11)
*P* value			0.5884	0.2139
After 90 d				
*N*	2,131	2,117	2,104	2,119
Patient deaths, *n* (%)	27 (1.3)	41 (1.9)	41 (1.9)	31 (1.5)
BGF 320/18/9.6 μg vs. comparators				
Hazard ratio (95% CI)	—	0.63 (0.39–1.03)	0.63 (0.38–1.02)	0.83 (0.49–1.39)
*P* value		0.0644	0.0618	0.4750
BGF 160/18/9.6 μg vs. comparators				
Hazard ratio (95% CI)	—	—	1.00 (0.65–1.54)	1.32 (0.83–2.10)
*P* value			0.9837	0.2501

*Definition of abbreviations*: BFF = budesonide/formoterol fumarate; BGF = budesonide/glycopyrrolate/formoterol fumarate; CI = confidence interval; GFF = glycopyrrolate/formoterol fumarate; ITT = intent-to-treat.

### Causes of Death and Characteristics of Patients Who Died

Overall, 164 on- and off-treatment deaths were reported in the original dataset, compared with 202 deaths in the final retrieved dataset (Table 2). Of the deaths in the final dataset, 156 were adjudicated; of these, 67 were attributed to cardiovascular causes (0.5%, 0.8%, 1.4%, and 0.5% in the BGF 320, BGF 160, GFF, and BFF groups, respectively) and 34 were attributed to respiratory causes (0.3%, 0.6%, 0.4%, and 0.3%, respectively).

**Table 2.** Summary of On- and Off-Treatment Deaths (ITT Population)

		BGF 320/18/9.6 μg (*N = 2,137*)	BGF 160/18/9.6 μg (*N = 2,121*)	GFF 18/9.6 μg (*N = 2,120*)	BFF 320/9.6 μg (*N = 2,131*)	All Patients (*N = 8,509*)
Total deaths*						
Original dataset		30 (1.4)	44 (2.1)	52 (2.5)	38 (1.8)	164 (1.9)
Final retrieved dataset		37 (1.7)	55 (2.6)	64 (3.0)	46 (2.2)	202 (2.4)
Deaths included in the time-to-death analyses^†^						
Original dataset		28 (1.3)	39 (1.8)	49 (2.3)	34 (1.6)	150 (1.8)
Final retrieved dataset		30 (1.4)	44 (2.1)	56 (2.6)	40 (1.9)	170 (2.0)
Adjudicated deaths^‡^						
Original dataset		27 (1.3)	42 (2.0)	47 (2.2)	35 (1.6)	151 (1.8)
Final retrieved dataset		28 (1.3)	43 (2.0)	50 (2.4)	35 (1.6)	156 (1.8)^§^
Cardiovascular		11 (0.5)	16 (0.8)	29 (1.4)	11 (0.5)	67 (0.8)
Respiratory		7 (0.3)	13 (0.6)	8 (0.4)	6 (0.3)	34 (0.4)
COPD		5 (0.2)	7 (0.3)	5 (0.2)	5 (0.2)	22 (0.3)
Pneumonia		2 (<0.1)	3 (0.1)	3 (0.1)	1 (<0.1)	9 (0.1)
Other respiratory		0	3 (0.1)	0	0	3 (<0.1)
Cancer		2 (<0.1)	6 (0.3)	3 (0.1)	7 (0.3)	18 (0.2)
Other		8 (0.4)	8 (0.4)	10 (0.5)	11 (0.5)	37 (0.4)
Nonadjudicated deaths (all-cause)						
Original dataset		3 (0.1)	2 (<0.1)	5 (0.2)	3 (0.1)	13 (0.2)
Final retrieved dataset		9 (0.4)	12 (0.6)	14 (0.7)	11 (0.5)	46 (0.5)

*Definition of abbreviations*: BFF = budesonide/formoterol fumarate; BGF = budesonide/glycopyrrolate/formoterol fumarate; COPD = chronic obstructive pulmonary disease; GFF = glycopyrrolate/formoterol fumarate; ITT = intent-to-treat.

Data are *n* (%).

* Includes all reported deaths occurring at any time after the first dose of treatment, without restriction as to how late the death was observed.

^†^ Includes deaths up to and including the Week 52 visit.

^‡^ Only deaths that were associated with at least one serious adverse event were adjudicated (i.e., vital status of death without a known associated adverse event was not adjudicated).

^§^ The five additional causes of death adjudicated in the final retrieved dataset were as follows: cardiovascular, *n* = 1 (in the GFF group); other, *n* = 4 (two in the GFF group and one each in the BGF 320/18/9.6 μg and BGF 160/18/9.6 μg groups).

The baseline characteristics of patients who died or survived are shown in Tables E4 and E5. Overall, compared with patients who survived, the patients who died had a higher mean age and a lower mean FEV_1_% predicted and percentage reversibility, and a higher proportion reported prior ICS use (Table E4).

### Relationship between COPD Exacerbations and Mortality

Exacerbation rates were analyzed according to vital status at Day 365 to assess the relationship between COPD exacerbations and mortality. The rates of moderate or severe exacerbations and severe exacerbations were higher in patients who died (2.20 and 0.80 per yr, respectively) than in those who did not die (1.11 and 0.16 per yr) (Table E6).

Of the 134 patients who died on treatment, 76 (56.7%) had experienced a moderate or severe exacerbation, and 43 (32.1%) had experienced a severe exacerbation (Table E7). For those with moderate or severe exacerbations, the median time from exacerbation to death was 30 days (range, 0–239 d). For severe exacerbations, the median time from exacerbation to death was 19 days (range, 0–278 d). Among the patients who died on treatment in the BGF 320 group, 36.0% and 60.0% had not experienced a moderate or severe exacerbation or severe exacerbation, respectively. In comparison, of the patients who died on treatment in the GFF group, 57.8% and 82.2% had not experienced moderate or severe exacerbations or severe exacerbations, respectively.

## Discussion

In the 52-week ETHOS trial, triple therapy with BGF 320 reduced the risk of death versus GFF and numerically reduced risk versus BFF in patients with moderate to very severe COPD and a history of exacerbations (7). Here, we presented analyses of the final dataset, including additional retrieved vital status data, which demonstrated that these findings were robust and were not solely, or even primarily, due to an acute ICS withdrawal effect. Furthermore, adjudicated causes of death and results for the median time from exacerbation to death suggest a potential role for ICS in mortality that may not be directly related to effects on COPD exacerbations.

In the final dataset, the risk of death (on and off treatment) with BGF 320 was 49% lower than that with GFF, which was consistent with the 46% reduction observed in the original dataset (7). Similarly, the 28% risk reduction for BGF 320 versus BFF was consistent with the 22% risk reduction in the original dataset. These results were robust to missing data, as demonstrated by tipping-point analyses in the final dataset that showed that the postwithdrawal hazard rate for BGF 320 would have to be more than 225 times the observed hazard rate during the study to lose significance versus GFF. Furthermore, the continued separation of the BGF 320 and GFF arms in the Kaplan-Meier curves demonstrates that the benefit was sustained throughout the treatment period. Notably, the benefit of BGF 320 versus GFF was shown across all subgroups of prior exacerbation history (moderate or severe, and severe) and baseline post-bronchodilator FEV_1_% predicted, with numerically larger effect sizes in patients with two or more exacerbations in the previous year or a post-bronchodilator FEV_1_ ≥50% predicted. These subgroups had substantial overlap because of the inclusion criteria requirement for a prior exacerbation history of ≥2 moderate or ≥1 severe exacerbations in patients with an FEV_1_ ≥50% of predicted. The treatment difference for BGF 320 versus GFF was also influenced by eosinophil count, with the benefit generally increasing above eosinophil counts of ∼200 cells/mm^3^. Overall, the subgroup analyses suggest that individual patient characteristics may affect the magnitude of the treatment benefit observed with triple therapy, although these findings should be interpreted with caution because of the small size of some subgroups.

Importantly, the reduction in mortality for BGF 320 relative to GFF is consistent with the benefit observed in a similar population of patients with COPD in the 52-week IMPACT trial, in which the risk of all-cause mortality with fluticasone furoate/umeclidinium/vilanterol (ICS/LAMA/LABA) relative to that of umeclidinium/vilanterol (LAMA/LABA) was 42% lower for on-treatment deaths and 28% lower for on- and off-treatment deaths after inclusion of final vital status data (6, 10). Also, a pooled analysis of three 52-week trials evaluating triple therapy with beclometasone dipropionate/glycopyrrolate/formoterol fumarate (TRIBUTE, TRINITY, and TRILOGY) suggested that patients who received triple therapy had a 28% lower risk of death than those who received the LAMA tiotropium or the LAMA/LABA glycopyrrolate/indacaterol; however, statistical significance was not reached (11). In interpreting findings across these studies, it is important to consider ICS dose potency. In this regard, the relative potency of the fluticasone furoate and beclometasone propionate doses used in these studies is most comparable with the 320-μg budesonide dose of BGF (12). Furthermore, although the comparison between the two doses of BGF was not part of the prespecified testing hierarchy in ETHOS, the HR of 0.66 (95% CI, 0.41–1.05) for BGF 320 versus BGF 160 suggests that the ICS effect was dose dependent in this patient population.

The annual absolute risk reductions in all-cause mortality for triple therapy versus LAMA/LABA were −1.24% in ETHOS (for BGF 320 vs. GFF), and −0.83% in IMPACT (for fluticasone furoate/umeclidinium/vilanterol vs. umeclidinium/vilanterol) (10). Both ETHOS and IMPACT enrolled patients at a high risk of COPD exacerbations, with the majority having two or more exacerbations in the previous year. This may have contributed to the larger ICS benefit observed in these studies compared with the TORCH (Towards a Revolution in COPD Health) and SUMMIT (Study to Understand Mortality and Morbidity in COPD) trials, which did not require patients to have a recent exacerbation history and reported nonsignificant reductions in all-cause mortality for ICS/LABA versus placebo (4, 5, 13). The nonsignificant risk reduction observed in ETHOS with BGF 320 relative to BFF also suggests a benefit of the LAMA component of triple therapy. This is consistent with the results of the UPLIFT (Understanding Potential Long-Term Impacts on Function with Tiotropium) study, in which tiotropium significantly reduced mortality compared with placebo during the 4 years of treatment (3).

Although exacerbations, particularly severe exacerbations, are known to be a risk factor for COPD mortality (14, 15), the mortality benefits that we observed in ETHOS cannot be explained solely by benefits in exacerbations, as both doses of BGF showed comparable effects in reducing moderate or severe exacerbation and severe exacerbation rates versus both dual therapies (7). Also, 42.4% of the patients who died in ETHOS had not experienced a moderate or severe exacerbation during the study, indicating that other factors were involved. In this regard, it is notable that the most common adjudicated cause of death in ETHOS (as in IMPACT) was cardiovascular, and both studies reported fewer deaths due to cardiovascular causes in the ICS groups compared with the LAMA/LABA group (6). It is also interesting to note that the number of nonfatal myocardial infarction events in ETHOS was lower in the BGF 320 and BFF arms relative to the GFF arm, although the total number of events was low (7). The prevalence of significant cardiovascular conditions in ETHOS was balanced across groups at baseline, suggesting that these treatment differences were not driven by an imbalance in risk. Although the SUMMIT trial found that ICS/LABA treatment did not reduce cardiovascular events versus placebo in patients with high risk of cardiovascular disease (5), all patients in SUMMIT had moderate COPD, and the majority did not report an exacerbation in the previous year or ICS use at study entry, which may have contributed to the difference in findings.

Benefits of ICS treatment on cardiovascular outcomes have also been observed in several other studies. Although the precise mechanisms for these effects remain unclear, ICS may reduce systemic inflammation and reduce hyperinflation, leading to improved cardiac function (16–18). Accordingly, a *post hoc* analysis of the EUROSCOP (European Respiratory Society Study on Chronic Obstructive Pulmonary Disease) study showed that the incidence of ischemic cardiac events was significantly lower in patients with COPD treated with 800 μg of budesonide compared with those who received placebo (19). Similarly, an observational study of all-cause mortality 90–365 days after hospital discharge for a COPD exacerbation showed that ICS use was associated with a 25% reduction in the risk of death, primarily because of fewer cardiovascular deaths (20). Furthermore, a pooled analysis of three 52-week phase III studies found that patients with COPD who received a beclometasone-containing treatment (ICS/LABA or ICS/LAMA/LABA) had a lower risk of nonrespiratory death than patients who did not receive beclometasone (LAMA or LAMA/LABA), whereas there was no difference in the risk of respiratory death (11). Although these studies are not directly comparable because of differences in design and patient populations, they provide supportive evidence for the mortality results in ETHOS and IMPACT, indicating that these are not chance findings.

It has previously been suggested that the benefits of triple therapy on mortality may represent an effect of acute withdrawal of therapy at randomization, including ICS withdrawal (21). To address these concerns, we assessed the potential impact of treatment withdrawal in several different ways. The first approach was to perform a subgroup analysis by prior treatment with triple therapy. As expected, the benefits of triple therapy versus dual therapies were larger in patients who were receiving triple therapy at study entry than in those who were not. However, as numerical benefits were observed in both subgroups, this finding did not appear to be solely driven by acute stepdown withdrawal of the ICS or LAMA component.

To specifically assess the impact of ICS withdrawal, additional analyses were undertaken. First, we examined treatment differences in mortality among patients who did not enter the study on ICS. However, the number of deaths in this subgroup was small (*n* = 5–8 across treatment groups), and a benefit of BGF 320 versus dual therapies on mortality could not be determined. The second approach was to perform landmark analyses excluding the first 30, 60, or 90 days of treatment (for the full population and for the subgroup of patients who entered the study on ICS). The results of these landmark analyses suggested that the benefits of triple therapy were not due to acute ICS withdrawal, as they were similar to those of the full analysis. The majority of deaths occurred after the first 90 days of treatment, and the HRs after exclusion of the first 90 days remained ≤0.63, even among the subgroup of patients who entered the study on ICS. It is important to note that in the Kaplan-Meier plot of time to death for the full analysis, the curves for BFF and GFF were similar for the first 9 weeks of the study, which also argues against an acute ICS withdrawal effect. Lastly, the HR for BGF 320 versus GFF remained relatively stable throughout the duration of the study when all deaths before each time point were excluded; this suggests a persistent effect even throughout the second half of the treatment period, in which the impact of acute ICS withdrawal would have been anticipated to be minimal. Overall, our findings suggest that there are benefits of both the ICS and LAMA components of triple therapy in modulating the long-term risk of mortality. It is important to recognize that ETHOS was not designed to assess ICS or LAMA withdrawal (unlike other trials such as WISDOM (Withdrawal of Inhaled Steroids during Optimised Bronchodilator Management) and SUNSET (Study to Understand the Safety and Efficacy of ICS Withdrawal from Triple Therapy in COPD), which evaluated a gradual stepdown of ICS treatment) (22, 23). We cannot, therefore, exclude the possibility that the discontinuation of therapy may have contributed to some of the early death events, either through withdrawal effects or simply by removing the benefits of therapy; however, the findings presented here suggest that the overall results for mortality cannot be explained solely by acute treatment withdrawal.

In conclusion, the results of ETHOS support the benefit of triple therapy with BGF 320 in reducing all-cause mortality versus a LAMA/LABA (GFF) in patients with moderate to very severe COPD and a history of exacerbations. These benefits were robust to missing data and do not appear to be driven by acute ICS withdrawal. Although additional studies are required to characterize the mechanism of this effect further, a reduction in deaths from cardiovascular causes appeared to account for the majority of the treatment difference. Regardless of the precise mechanism, our findings underscore the need to target mortality reduction as an achievable goal in the treatment of COPD, which is a leading cause of death worldwide.

## Supplementary Material

Supplements

Author disclosures
