# Supplementary material for: Reduced All-Cause Mortality in the ETHOS Trial of Budesonide/Glycopyrrolate/Formoterol for Chronic Obstructive Pulmonary Disease. A Randomized, Double-Blind, Multicenter, Parallel-Group Study
Source: Am J Respir Crit Care Med. 2021 Mar 1;203(5):553–64. doi: 10.1164/rccm.202006-2618OC (PMC7924571; doi:10.1164/rccm.202006-2618OC)
Supplement: Supplements [file rccm.202006-2618OC.html]

Reduced All-Cause Mortality in the ETHOS Trial of Budesonide/Glycopyrrolate/Formoterol for Chronic Obstructive Pulmonary Disease. A Randomized, Double-Blind, Multicenter, Parallel-Group Study | American Journal of Respiratory and Critical Care Medicine

- disclosures.pdf (316 KB)
- martinez\_data\_supplement.pdf (1 MB)
