# Supplementary material for: Reduced All-Cause Mortality in the ETHOS Trial of Budesonide/Glycopyrrolate/Formoterol for Chronic Obstructive Pulmonary Disease. A Randomized, Double-Blind, Multicenter, Parallel-Group Study
Source: Am J Respir Crit Care Med. 2021 Mar 1;203(5):553–64. doi: 10.1164/rccm.202006-2618OC (PMC7924571; doi:10.1164/rccm.202006-2618OC)
Supplement: Supplements [file rccm.202006-2618OC_martinez_data_supplement.pdf]

Reduced All-Cause Mortality in the ETHOS Trial of Budesonide/Glycopyrrolate/Formoterol for COPD: A Randomized, Double-Blind, Multi-Center Parallel-Group Study

Fernando J. Martinez, Klaus F. Rabe, Gary T. Ferguson, Jadwiga A. Wedzicha, Dave Singh, Chen Wang, Kimberly Rossman, Earl St. Rose, Roopa Trivedi, Shaila Ballal, Patrick Darken, Magnus Aurivillius, Colin Reisner, and Paul Dorinsky, on behalf of the ETHOS investigators

Online Data Supplement

Methods

For budesonide/glycopyrrolate/formoterol fumarate metered dose inhaler (BGF) 320/18/9.6 µg, BGF 160/18/9.6 µg, glycopyrrolate/formoterol fumarate 18/9.6 µg, and budesonide/formoterol fumarate 320/18/9.6 µg, the doses of 18 µg glycopyrrolate and 9.6 µg formoterol fumarate are equivalent to 14.4 µg of glycopyrronium and 10 µg of formoterol fumarate dihydrate, respectively.

Screening and Washout Procedures

After the first screening visit, eligible patients had their chronic obstructive pulmonary disease (COPD) maintenance therapy adjusted as follows:

- Patients discontinued the use of short-acting muscarinic antagonists, short-acting β_2-agonists, long-acting muscarinic antagonists, and/or long-acting β_2-agonists for the duration of the study and were switched to ipratropium bromide 4 times daily, and albuterol as needed (up to 4 times per day) for control of symptoms during the screening period.
- Patients receiving a maintenance dose of an inhaled corticosteroid (ICS) as part of a combination therapy, who had been on the ICS component (and maintained on a stable dose) for at least 4 weeks prior to Visit 1, received the corresponding dose of ICS administered as a single agent, in addition to ipratropium bromide and albuterol.

All adjusted maintenance therapy for COPD, including ICS, was stopped at randomization.

52-Week, Post-Randomization Vital Status Confirmation

All patients who discontinued study treatment prior to 52 weeks post-randomization had their vital status confirmed at 52 weeks post-randomization. The following attempts were made, if appropriate, to confirm the vital status and cause of death:

- The first and second attempts were to be conducted as telephone follow-up calls to the patient within 2 weeks after 52 weeks post-randomization.
- The third attempt was by certified mail to the patient's address provided at the time of informed consent within 3 weeks after 52 weeks post-randomization.
- The fourth attempt was made as a telephone follow-up call to the next of kin/emergency contact provided at the time of informed consent within 4 weeks after 52 weeks post-randomization.
- A fifth attempt was made through a certified letter to the next of kin/emergency contact provided at the time of informed consent within 5 weeks after 52 weeks post-randomization.
- After the fifth attempt, the study site was to contact the national death registries (if available in that country) to confirm the date and cause of death.

Figure E1. Tipping point analysis one on the comparison of BGF 320/18/9.6 µg versus GFF 18/9.6 µg for time to all-cause death (final retrieved dataset; ITT population).

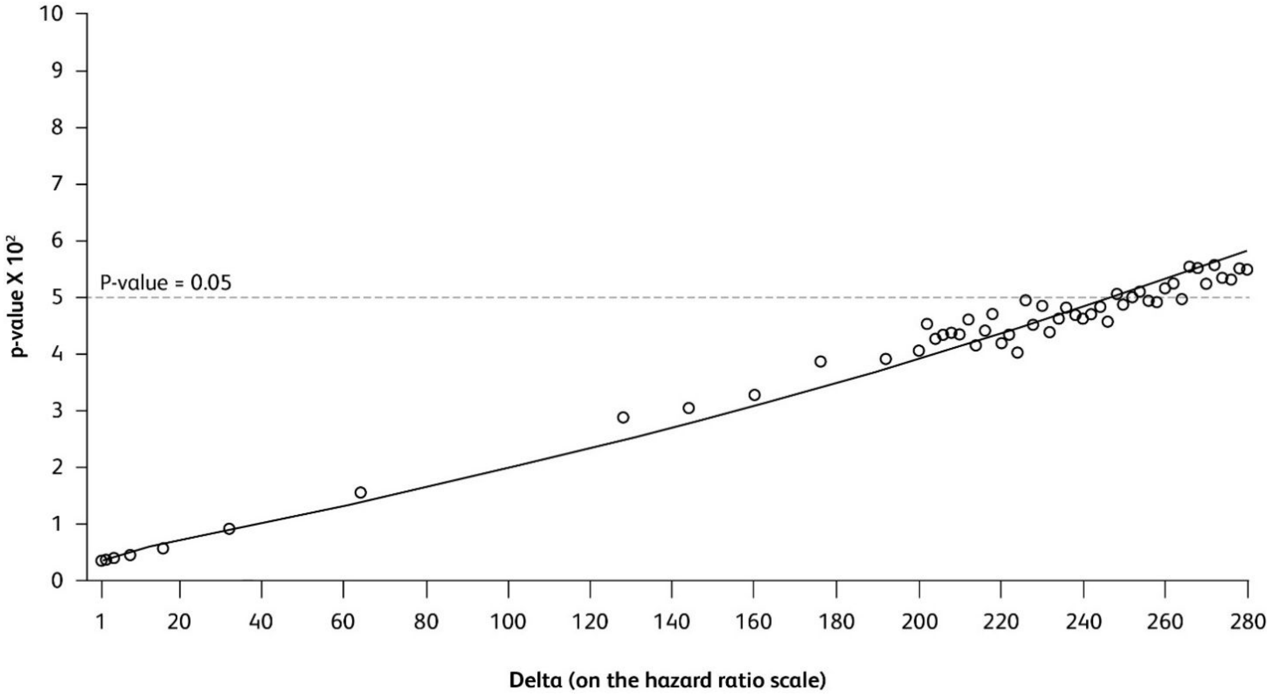

All non-BGF patients with missing vital status at Week 52 were censored at Week 52. All BGF 320/18/9.6 µg patients with missing vital status at Week 52 were imputed using the BGF 320/18/9.6 µg hazard rate. Delta is defined as the amount by which the BGF-arm hazard rate was increased. A delta of 1 equates to no difference from the observed data model imputed value. Delta is applied multiplicatively on the hazard rate. The ITT population included all patients who were randomized to treatment and received any amount of study drug.

Definitions of abbreviations: BGF = budesonide/glycopyrrolate/formoterol fumarate; GFF = glycopyrrolate/formoterol fumarate; ITT = intent-to-treat.

Figure E2. Tipping point analysis two on the comparison of BGF 320/18/9.6 µg versus GFF 18/9.6 µg for time to all-cause death (final retrieved dataset; ITT population).

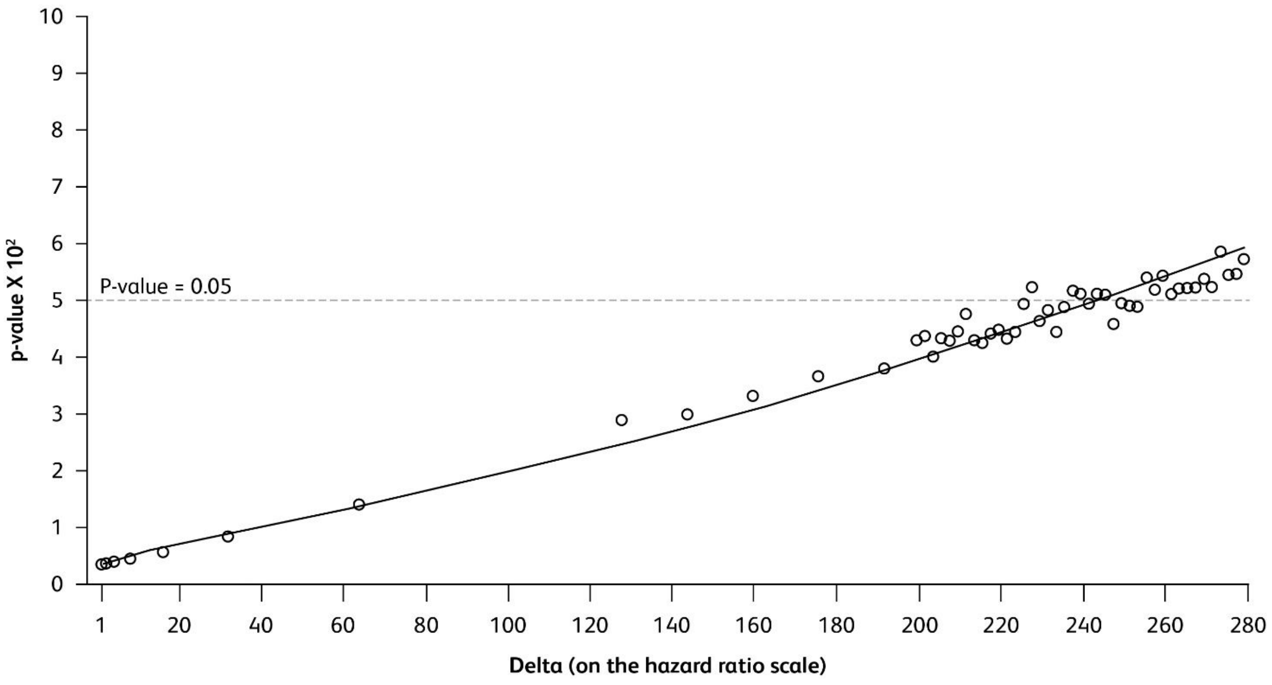

All patients with missing vital status at Week 52 were imputed using the hazard rate for their respective treatment arm. Delta is defined as the amount by which the respective BGF 320/18/9.6 µg hazard rate was increased. A delta of 1 equates to no difference from the observed data model imputed value. Delta is applied multiplicatively on the hazard rate.

Definitions of abbreviations: BGF = budesonide/glycopyrrolate/formoterol fumarate; GFF = glycopyrrolate/formoterol fumarate; ITT = intent-to-treat.

Figure E3. Incidence of death by blood eosinophil count (final retrieved dataset; ITT population).

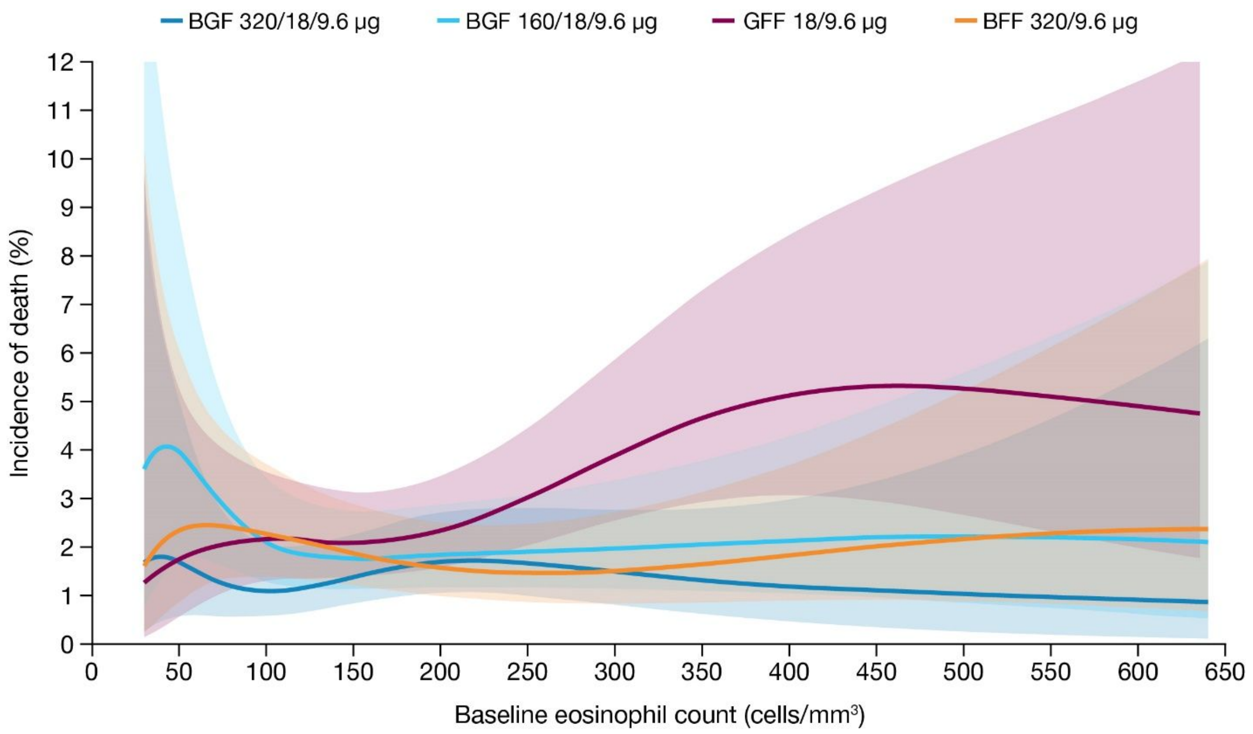

Data from generalized additive model. Banded areas indicate 95% confidence intervals that reflect the skewed distribution of eosinophil counts, i.e., 17.3% of patients had counts <100 cells/mm^3, 67.9% had 100–300 cells/mm^3, and 14.7% had >300 cells/mm^3.

Definitions of abbreviations: BFF = budesonide/formoterol fumarate; BGF = budesonide/glycopyrrolate/formoterol fumarate; GFF = glycopyrrolate/formoterol fumarate; ITT = intent-to-treat.

Figure E4. Kaplan–Meier plot for time to all-cause death excluding the first 90 days in patients using ICS at study entry (final retrieved dataset; ITT population).

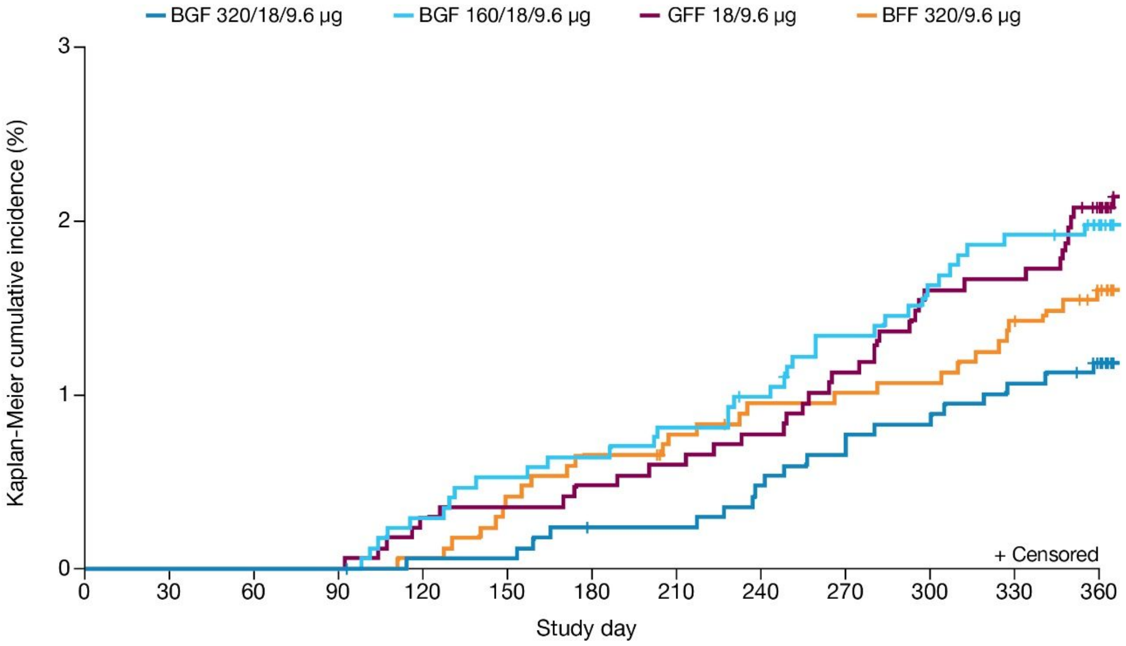

Patients at risk										
BGF 320/18/9.6 µg	1691	1689	1689	1685	1685	1681	1678	1675	1671	1666
BGF 160/18/9.6 µg	1718	1713	1709	1707	1704	1699	1682	1687	1682	1678
GFF 18/9.6 µg	1683	1678	1677	1675	1673	1670	1664	1656	1655	1645
BFF 320/9.6 µg	1685	1684	1678	1674	1670	1666	1665	1664	1658	1649

Definitions of abbreviations: BFF = budesonide/formoterol fumarate;

BGF = budesonide/glycopyrrolate/formoterol fumarate; GFF = glycopyrrolate/formoterol fumarate;

ITT = intent-to-treat.

Figure E5. Hazard ratio for BGF 320/18/9.6 µg versus GFF over time, excluding all previous data (final retrieved dataset; ITT population).

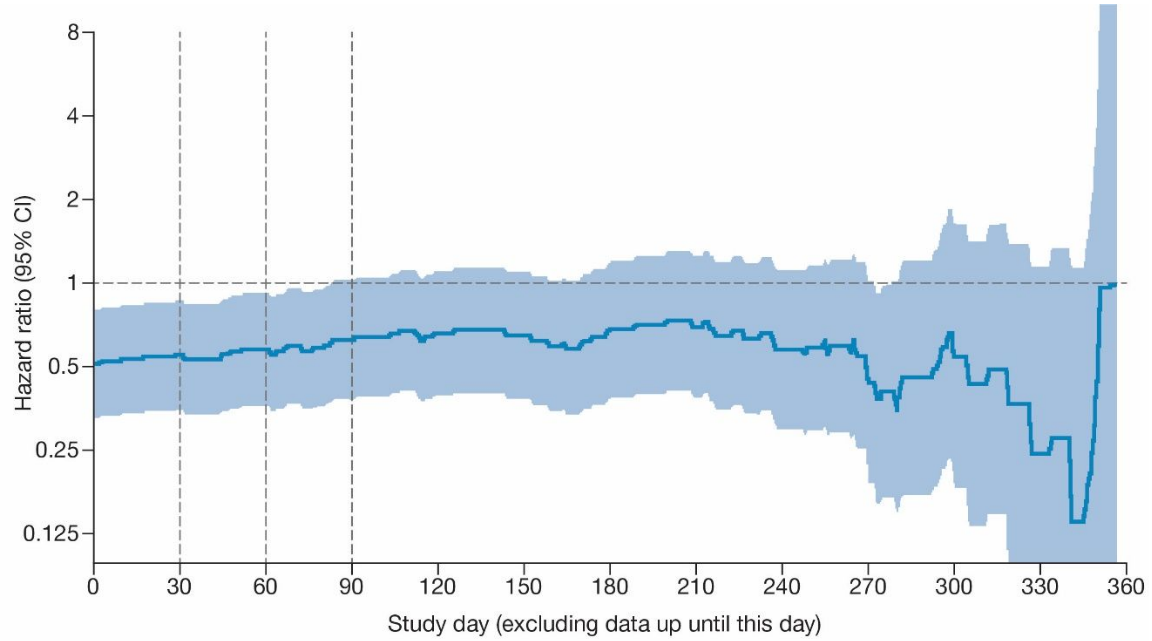

Patients at risk													
BGF 320/18/9.6 µg	2137	2136	2134	2131	2127	2126	2122	2120	2114	2107	2104	2100	2089
GFF 18/9.6 µg	2120	2116	2112	2104	2098	2097	2090	2088	2085	2078	2069	2068	2056

Definitions of abbreviations: BGF = budesonide/glycopyrrolate/formoterol fumarate; CI = confidence interval; GFF = glycopyrrolate/formoterol fumarate; ITT = intent-to-treat.

Table E1. Baseline Cardiovascular Medical History (Safety Population)

	**BGF 320/18/9.6 µg (*N* = 2,144)**	**BGF 160/18/9.6 µg (*N* = 2,124)**	**GFF 18/9.6 µg (*N* = 2,125)**	**BFF 320/9.6 µg (*N* = 2,136)**	**All patients (*N* = 8,529)**
≥1 Cardiovascular risk factor of interest	1,511 (70.5)	1,505 (70.9)	1,492 (70.2)	1,513 (70.8)	6,021 (70.6)
Hypertension	1,272 (59.3)	1,252 (58.9)	1,250 (58.8)	1,251 (58.6)	5,025 (58.9)
High total cholesterol	769 (35.9)	769 (36.2)	744 (35.0)	761 (35.6)	3,043 (35.7)
Diabetes	398 (18.6)	400 (18.8)	339 (16.0)	358 (16.8)	1,495 (17.5)
Angina	162 (7.6)	154 (7.3)	147 (6.9)	165 (7.7)	628 (7.4)
Myocardial infarction	142 (6.6)	166 (7.8)	138 (6.5)	158 (7.4)	604 (7.1)
Peripheral vascular disease	162 (7.6)	151 (7.1)	137 (6.4)	116 (5.4)	566 (6.6)
Atrial fibrillation	121 (5.6)	92 (4.3)	89 (4.2)	87 (4.1)	389 (4.6)
Stroke	71 (3.3)	54 (2.5)	51 (2.4)	65 (3.0)	241 (2.8)
Transient ischemic attack	40 (1.9)	47 (2.2)	52 (2.4)	36 (1.7)	175 (2.1)

Data are *n* (%).

Definitions of abbreviations: BFF = budesonide/formoterol fumarate;

BGF = budesonide/glycopyrrolate/formoterol fumarate; GFF = glycopyrrolate/formoterol fumarate.

Table E2. Time to On-Treatment Death (All-Cause) Using 7-Day Cut-Off (Final Retrieved Dataset; ITT Population)

	**BGF 320/18/9.6 µg (*N* = 2,137)**	**BGF 160/18/9.6 µg (*N* = 2,121)**	**GFF 18/9.6 µg (*N* = 2,120)**	**BFF 320/9.6 µg (*N* = 2,131)**
Patient deaths, *n* (%)	16 (0.7)	27 (1.3)	33 (1.6)	23 (1.1)
BGF 320/18/9.6 µg versus comparators				
Hazard ratio (95% CI)	—	0.59 (0.32–1.10)	0.45 (0.25–0.82)	0.66 (0.35–1.25)
P-value		0.0957	0.0086	0.2050
BGF 160/18/9.6 µg versus comparators				
Hazard ratio (95% CI)	—	—	0.76 (0.46–1.26)	1.12 (0.64–1.95)
P-value			0.2902	0.6906

For this analysis, deaths were considered to be on-treatment if they occurred within 7 days of the last day of treatment.

Definitions of abbreviations: BFF = budesonide/formoterol fumarate; BGF = budesonide/glycopyrrolate/formoterol fumarate; CI = confidence interval; GFF = glycopyrrolate/formoterol fumarate; ITT = intent-to-treat.

Table E3. Time to Death (All-Cause) Occurring After the First 30, 60, or 90 Days of Treatment in Patients Receiving ICS at Study Entry (Final Retrieved Dataset; ITT Population)

	**BGF 320/18/9.6 µg (*N* = 1,696)**	**BGF 160/18/9.6 µg (*N* = 1,722)**	**GFF 18/9.6 µg (*N* = 1,698)**	**BFF 320/9.6 µg (*N* = 1,694)**
**After 30 days**				
N	1,695	1,722	1,694	1,690
Patient deaths, *n* (%)	22 (1.3)	37 (2.1)	47 (2.8)	31 (1.8)
BGF 320/18/9.6 µg versus comparators				
Hazard ratio (95% CI)	—	0.57 (0.33–0.98)	0.45 (0.27–0.75)	0.67 (0.38–1.16)
P-value		0.0400	0.0022	0.1550
BGF 160/18/9.6 µg versus comparators				
Hazard ratio (95% CI)	—	—	0.79 (0.51–1.21)	1.17 (0.73–1.89)
P-value			0.2696	0.5134
**After 60 days**				
N	1,694	1,721	1,690	1,687
Patient deaths, *n* (%)	22 (1.3)	36 (2.1)	43 (2.5)	28 (1.7)
BGF 320/18/9.6 µg versus comparators				
Hazard ratio (95% CI)	—	0.59 (0.34–1.01)	0.49 (0.29–0.82)	0.74 (0.42–1.31)
P-value		0.0522	0.0072	0.2990
BGF 160/18/9.6 µg versus comparators				
Hazard ratio (95% CI)	—	—	0.83 (0.54–1.30)	1.26 (0.77–2.07)
P-value			0.4203	0.3543
**After 90 days**				
N	1,691	1,718	1,683	1,685
Patient deaths, *n* (%)	20 (1.2)	34 (2.0)	36 (2.1)	27 (1.6)
BGF 320/18/9.6 µg versus comparators				
Hazard ratio (95% CI)	—	0.56 (0.32–0.98)	0.53 (0.30–0.92)	0.69 (0.39–1.25)
P-value		0.0438	0.0242	0.2229
BGF 160/18/9.6 µg versus comparators				
Hazard ratio (95% CI)	—	—	0.94 (0.59–1.50)	1.24 (0.75–2.05)
P-value			0.7970	0.4099

Definitions of abbreviations: BFF = budesonide/formoterol fumarate;

BGF = budesonide/glycopyrrolate/formoterol fumarate; CI = confidence interval;

GFF = glycopyrrolate/formoterol fumarate; ITT = intent-to-treat.

Table E4. Demographic and Baseline Characteristics by Vital Status (Final Retrieved Dataset; ITT Population).

	**Patients who died (N=170)**	**Patients who survived (N=8,339)**
Mean age, years (SD)	67.7 (7.7)	64.6 (7.6)
Male, n (%)	120 (70.6)	4,961 (59.5)
Race, n (%)		
White	147 (86.5)	7,079 (84.9)
Asian	8 (4.7)	643 (7.7)
Black	5 (2.9)	300 (3.6)
American Indian/Alaska Native	5 (2.9)	137 (1.6)
Other	5 (2.9)	180 (2.1)
Current smoker, n (%)	63 (37.1)	3,432 (41.2)
Mean number of pack-years smoked (SD)	52.9 (30.5)	47.5 (25.8)
Exacerbation history in the past 12 months		
1	84 (49.4)	3,615 (43.4)
≥2	86 (50.6)	4,724 (56.6)
Blood eosinophil count		
Median (range)	175 (35–685)	165 (0–2510)
≥150 cells/mm^3	103 (60.6)	4,998 (59.9)
≥300 cells/mm^3	29 (17.1)	1,225 (14.7)
ICS use at screening, n (%)	144 (84.7)	6,702 (80.4)
Mean post-bronchodilator FEV_1, % predicted at screening (SD)	40.1 (10.4)	43.4 (10.3)
% Reversibility to albuterol (SD)	13.0 (13.4)	15.5 (15.8)
Mean CAT score (SD)	20.4 (6.7)	19.6 (6.5)

Definitions of abbreviations: CAT = COPD Assessment Test; COPD = chronic obstructive pulmonary disease; FEV_1 = forced expiratory volume in 1 second; ITT = intent-to-treat; SD = standard deviation.

Table E5. Demographic and Baseline Characteristics of Patients who Died (Final Retrieved Dataset; ITT Population).

	BGF 320/18/9.6 μg (N = 30)	BGF 160/18/9.6 μg (N = 44)	GFF 18/9.6 μg (N = 56)	BFF 320/9.6 μg (N = 40)
Mean age, years (SD)	69.4 (6.9)	67.3 (8.6)	66.5 (7.0)	68.5 (8.0)
Male, n (%)	26 (86.7)	25 (56.8)	38 (67.9)	31 (77.5)
Race, n (%)				
White	24 (80.0)	40 (90.9)	49 (87.5)	34 (85.0)
Asian	3 (10.0)	0	2 (3.6)	3 (7.5)
Black	2 (6.7)	1 (2.3)	1 (1.8)	1 (2.5)
American Indian/Alaska Native	1 (3.3)	1 (2.3)	3 (5.4)	0
Other	0	2 (4.5)	1 (1.8)	2 (5.0)
Current smoker, n (%)	12 (40.0)	17 (38.6)	22 (39.3)	12 (30.0)
Mean number of pack-years smoked (SD)	65.3 (43.5)	50.6 (24.3)	52.7 (25.7)	46.4 (29.6)
Exacerbation history in the past 12 months				
1	18 (60.0)	25 (56.8)	22 (39.3)	19 (47.5)
≥2	12 (40.0)	19 (43.2)	34 (60.7)	21 (52.5)
Blood eosinophil count				
Median (range)	185 (45–475)	158.3 (35–550)	202.5 (40–685)	152.5 (45–590)
≥150 cells/mm^3	22 (73.3)	25 (56.8)	35 (62.5)	21 (52.5)
≥300 cells/mm^3	3 (10.0)	6 (13.6)	13 (23.2)	7 (17.5)
ICS use at screening, n (%)	22 (73.3)	37 (84.1)	51 (91.1)	34 (85.0)
Mean post-bronchodilator FEV_1, % predicted at screening (SD)	39.3 (8.2)	38.9 (10.3)	41.5 (10.1)	40.2 (12.5)
% Reversibility to albuterol (SD)	9.5 (12.2)	12.1 (12.5)	14.0 (13.4)	15.3 (15.0)
Mean CAT score (SD)	20.4 (6.3)	20.0 (5.9)	21.2 (7.3)	19.5 (6.9)

Includes all deaths up to and including the Week 52 visit.

Definitions of abbreviations: BFF = budesonide/formoterol fumarate; BGF = budesonide/glycopyrrolate/formoterol fumarate; FEV_1 = forced expiratory volume in 1 second; GFF = glycopyrrolate/formoterol fumarate; ITT = intent-to-treat; SD = standard deviation.

Table E6. Rates of Moderate or Severe COPD Exacerbations According to Vital Status at Day 365 (Final Retrieved Dataset; ITT Population).

	Moderate or severe exacerbations	Severe exacerbations
**Patients who died (*N* = 170)**		
Patients with exacerbations, n (%)	98 (57.6)	50 (29.4)
Rate (per year)	2.20	0.80
**Patients who did not die (*N* = 8,339)**		
Patients with exacerbations, n (%)	4,234 (50.8)	991 (11.9)
Rate (per year)	1.11	0.16

Definitions of abbreviations: COPD = chronic obstructive pulmonary disease; ITT = intent-to-treat.

Table E7. Time from COPD Exacerbation to Death (Final Retrieved Dataset; mITT Population).

	**BGF 320/18/9.6 µg (*N* = 2,137)**	**BGF 160/18/9.6 µg (*N* = 2,121)**	**GFF 18/9.6 µg (*N* = 2,120)**	**BFF 320/9.6 µg (*N* = 2,131)**
**Patients who did not die**				
N	2,112	2,085	2,075	2,103
Had a moderate/severe exacerbation, n (%)	1,026 (48.6)	1,006 (48.2)	1,064 (51.3)	1,094 (52.0)
Had a severe exacerbation, n (%)	221 (10.5)	234 (11.2)	240 (11.6)	262 (12.5)
**Patients who died**				
N	25	36	45	28
Had a moderate/severe exacerbation	16 (64.0)	27 (75.0)	19 (42.2)	14 (50.0)
Died within 30 days of exacerbation start date, n (%)	9 (36.0)	11 (30.6)	12 (26.7)	6 (21.4)
Died within 60 days of exacerbation start date, n (%)	11 (44.0)	16 (44.4)	13 (28.9)	9 (32.1)
Median number of days from last exacerbation to death (range)	26 (0–214)	43 (1–238)	19 (4–239)	43 (1–202)
Had a severe exacerbation, n (%)	10 (40.0)	16 (44.4)	8 (17.8)	9 (32.1)
Died within 30 days of exacerbation start date, n (%)	7 (28.0)	9 (25.0)	8 (17.8)	5 (17.9)
Died within 60 days of exacerbation start date, n (%)	9 (36.0)	10 (27.8)	8 (17.8)	7 (25.0)
Median number of days from last exacerbation to death (range)	22 (0–106)	19 (1–238)	15 (4–21)	25 (1–278)

Analyses include all COPD exacerbations for patients who died on or before 30 days after the last dose of treatment.

Definitions of abbreviations: BFF = budesonide/formoterol fumarate; BGF = budesonide/glycopyrrolate/formoterol fumarate; COPD = chronic obstructive pulmonary disease; GFF = glycopyrrolate/formoterol fumarate; mITT = modified intent-to-treat.
